# Central fibrous area in the glomerular vascular pole consists of fibrous collagens and is associated with advanced age: a cross-sectional study

**DOI:** 10.1186/s12882-022-02835-2

**Published:** 2022-06-11

**Authors:** Satoshi Hara, Yutaka Yamaguchi, Takeshi Zoshima, Ichiro Mizushima, Kazunori Yamada, Ryo Inoue, Hisao Muto, Kazuaki Mizutomi, Masayoshi Hirata, Hideo Araki, Ryoichi Miyazaki, Mitsuhiro Kawano

**Affiliations:** 1 Department of Rheumatology, Kanazawa University Graduate School of Medicine, 13-1 Takaramachi, Kanazawa, Ishikawa Japan; 2 Medical Education Research Center, Graduate School of Medical Sciences, Kanazawa University, 13-1 Takaramachi, Kanazawa, Ishikawa Japan; 3 Yamaguchi’s Pathology Laboratory, 1-31-20Minoridai, Matsudo, Chiba, Japan; 4 Department of Internal Medicine, Saiseikai Kanazawa Hospital, Ni13-6 Akatsuchimachi, Kanazawa, Ishikawa Japan; 5 Department of Internal Medicine, Hokuriku Central Hospital, 123 Nodera, Oyabe, Toyama, Japan; 6 Department of Internal Medicine, Kaga Medical Center, Ri 36 Sakumimachi, Kaga, Ishikawa Japan; 7 Department of Internal Medicine, Takaoka City Hospital, 4-1 Takaramachi, Takaoka, Toyama, Japan; 8 Department of Nephrology and Rheumatology, Fukui Prefecture Hospital, 2-8-1 Yotsui, Fukui, Fukui Japan; 9 Department of Internal Medicine, Fujita Memorial Hospital, 4-15-7 Houei, Fukui, Fukui Japan

**Keywords:** Central fibrous area, Collagen, Older age, Myofibroblast

## Abstract

**Background:**

For the optimal management of patients with both allograft kidneys and native kidney diseases, the recognition of the histological features associated with older age is important. This is because most pathological findings are non-specific. Central fibrous areas (CFAs) have recently been proposed to be age-related. However, the components of CFAs and whether CFAs are observed in various kidney diseases remain undetermined. This cross-sectional study was undertaken to clarify the histological features, epidemiology, and clinicopathological features of CFAs.

**Methods:**

One hundred and one consecutive kidney needle biopsy specimens were retrospectively collected from seven facilities in the Hokuriku region and diagnosed at the Kanazawa University Hospital in 2015. First, the components of CFAs were analyzed using normal histostaining, immunostaining, and electron microscopy. Second, the patients were divided into two groups (CFA [+] or CFA [−]) according to the presence of CFA in the obtained samples. Clinical and histological features were compared between the two groups, and factors associated with CFA formation were determined using univariate and multivariate analyses. The number of CFAs per specimen was counted in the CFA (+) group. Third, the presence of myofibroblasts in CFA was examined by immunostaining.

**Results:**

CFAs were observed in 56 of 101 patients (55.4%) with various kidney diseases. CFAs consist of fibrillar collagens (collagen I and III) in addition to non-fibrillar collagens (collagen IV and VI), as confirmed by electron microscopy. Clinically, the CFA (+) group was older and had a significantly higher prevalence of hypertension and hyperlipidemia than the CFA (−) group. Histologically, elastofibrosis of the interlobular artery, arteriolar hyalinosis, and membranous nephropathy were significantly more evident in the CFA (+) group than in the CFA (−) group. Multivariate analysis revealed that older age was the sole factor associated with CFA formation. Finally, 27 of 58 (46.6%) CFA-containing glomeruli in 26 cases included alpha-smooth muscle actin-positive cells in or adjacent to the CFA.

**Conclusions:**

CFAs consist of fibrous collagens in addition to matrix collagens. CFA formation is associated with older age and was observed in various kidney diseases.

**Supplementary Information:**

The online version contains supplementary material available at 10.1186/s12882-022-02835-2.

## Background

Aging inevitably alters renal structure and function [1–5]. Histologically, the interlobular arteries of the kidney develop progressive arteriosclerosis, characterized by intimal and medial hypertrophy. This eventually leads to ischemic glomerular collapse, glomerulosclerosis, tubular atrophy, and interstitial fibrosis [1–6]. The glomerular filtration rate (GFR) declines with advancing age [1, 2, 4, 5]. In addition, aging increases susceptibility to renal injury, resulting in incomplete recovery and progressive kidney dysfunction [7–10]. Living-related kidney donors tend to be older, with an associated increase in delayed graft function and risk of acute rejection. These have the potential to adversely affect graft survival [5, 11–13]. Thus, recognizing the histological features associated with older age is helpful for the optimal management of patients with both native kidney diseases and allograft kidneys.

Recently, small, hyalinotic, and monotonous nodular areas in glomerular vascular poles lesions, called central fibrous areas (CFAs), have been detected as a valuable indicator of kidney aging [14]. CFAs have been observed in healthy kidneys and IgA nephropathy [14]. However, the components of CFAs and whether other kidney diseases conspire with CFAs remain unclear. To clarify these points, this study examined the histological features, epidemiology, and clinicopathological features of CFAs.

## Methods

### Study design

The present cross-sectional study used 107 consecutive kidney needle biopsy specimens of Japanese adult patients obtained from seven hospitals in the Hokuriku region (Kanazawa University Hospital, Saiseikai Kanazawa Hospital, Kaga Medical Center, Takaoka City Hospital, Hokuriku Hospital, Fukui Prefecture Hospital, and Fujita Memorial Hospital) who were diagnosed at Kanazawa University Hospital between January and December 2015. Kidney biopsies were performed when patients displayed kidney dysfunction or urinary abnormalities (proteinuria or hematuria). Among them, allograft biopsy (*n* = 2), renal amyloidosis (*n* = 2), or specimens containing fewer than five glomeruli (*n* = 2) were excluded, and the remaining 101 specimens were used in the present study. Background pathological diagnoses included: benign nephrosclerosis (*n* = 8), mesangial proliferative glomerulonephritis (IgA nephropathy, *n* = 20; IgA vasculitis, n = 2; non-IgA mesangial proliferative glomerulonephritis, *n* = 4), endocapillary proliferative glomerulonephritis (*n* = 2), lupus nephritis (*n* = 7), anti-neutrophil cytoplasmic autoantibody-associated vasculitis (*n* = 9), minimal change disease (*n* = 7), focal segmental glomerulosclerosis (primary, *n* = 0; secondary, *n* = 2), membranous nephropathy (*n* = 21), diabetic nephropathy (*n* = 7), acute tubulointerstitial nephritis and/or acute tubular injury (*n* = 8), minor glomerular abnormalities (*n* = 4), and others such as thrombotic microangiopathy (*n* = 2), thin basement membrane disease (*n* = 2), focal glomerular obsolescence (*n* = 2), smoking-related glomerulopathy (*n* = 1), acute pyelonephritis (*n* = 1), and glomerular hypertrophy (*n* = 1). Eight patients had two different kidney diseases.

CFA was defined as a periodic-acid Schiff (PAS)-weakly positive monotonous accumulation in the glomerular vascular pole. The nodular form was not essential in the present study unlike a previous study [14], because a CFA is often cylindrically shaped encircling afferent arteriole [15]. First, the components of a CFA were analyzed using normal histostaining, immunostaining, and electron microscopy. Second, the patients were divided into two groups (CFA [+] or CFA [−]) according to the presence of CFA in the obtained samples. Clinical and histological features were compared between the two groups, and factors associated with CFA formation were determined using univariate and multivariate analyses. The selected clinical parameters were as follows: age, sex, body mass index, hypertension, diabetes mellitus, hyperlipidemia, hyperuricemia, proteinuria, microscopic hematuria, serum creatinine level, and estimated GFR at the time of biopsy, which were collected from request forms for renal pathological diagnosis. The following histological parameters were selected: background pathological diagnosis, glomerular number, global glomerulosclerosis, segmental glomerulosclerosis, glomerular tuft size, elastofibrosis in the interlobular artery, arteriolar hyalinosis, tubular atrophy, and interstitial fibrosis.

### Histochemistry and immunohistochemistry

Samples were fixed in 10% formaldehyde or Bouin’s solution for paraffin blocks and 2% glutaraldehyde for transmission electron microscopy. Histostaining included PAS, periodic acid silver methenamine, Masson’s trichrome, and Congo red stains. The following specific primary antibodies were used for immunostaining: monoclonal mouse anti-human collagen I (clone COL-1; 1:100; Abcam, Cambridge, UK), polyclonal rabbit anti-human collagen III (1:400; Abcam), polyclonal rabbit anti-human collagen IV (1:200; Abcam), polyclonal rabbit anti-human collagen VI (1:100; Abcam), and monoclonal mouse anti-human alpha-smooth muscle actin (α-SMA) (clone 1A4; 1:200; Sigma-Aldrich, MO, USA). For immunostaining, antigens were retrieved by microwaves (10 mM citrate buffer; pH 6.0) for collagen I, or 100 μg/mL of proteinase K (Wako Pure Chemical Industries, Osaka, Japan) for collagen III, IV, VI, and α-SMA. The primary antibodies were then incubated in a biotinylated link (Dako), followed by a reaction with peroxidase-conjugated streptavidin (Dako). Peroxidase activity was visualized using liquid diaminobenzidine substrate (Dako). Hematoxylin was used to stain the nuclei. Elastofibrosis in the interlobular artery was characterized as mild, with vascular narrowing of up to 25% of the luminal area by fibrointimal thickening; moderate, with vascular narrowing of 26–50% of the luminal area; or severe, with vascular narrowing of > 50% of the luminal space. Arteriolar hyalinosis was graded as mild, with mild to moderate PAS-positive hyaline thickening in at least one arteriole; moderate, with moderate to severe PAS-positive hyaline thickening in at least one arteriole; or severe, with severe PAS-positive hyaline thickening in many arterioles. Tubular atrophy was classified as mild with involvement of up to 25% of the cortical tubule area, moderate with 26–50% involvement, and severe with > 50% involvement. Interstitial fibrosis was characterized as mild with involvement of 6–25% of the cortical area, moderate with 26–50% involvement, and severe with > 50% involvement. These grading systems were based on the Banff lesion grading system [16]. The CFA-positive ratio was calculated by dividing CFA numbers by glomerular numbers. The glomerular tuft area was calculated by using imaging software, NIS-Elements D version 4.20.00 (Nikon Solutions, Tokyo, Japan).

### Statistical analyses

The Mann–Whitney U test was performed for continuous variables between the two groups, and Pearson’s χ^2^ test was performed for binary variables between the two groups. Logistic regression analysis was performed for multivariate analysis. Variables for multivariate analysis were selected based on the results of the stepwise regression analysis. CFA numbers were compared with glomerular numbers obtained using linear regression analysis, and the coefficients of determination were calculated. IBM SPSS Statistics version 19 (International Business Machines Corporation, New Orchard, NY, USA) was used for the statistical analysis. Statistical significance was set at *p* < 0.05.

## Results

### Histological characteristics of a CFA in the glomerular vascular pole

One hundred and one kidney biopsy specimens obtained from 101 consecutive patients who did not meet exclusion criteria from seven hospitals during 2015 were included in the analyses. The clinical demographics was described in Table 1. The patients’ median age [interquartile range {IQR}] was 62.5 [55.3–71.0] years old. Median serum creatinine was 0.86 [IQR 0.66–1.17] mg/dL, and the median estimated GFR was 63.5 [IQR 39.0–83.7] mL/min/1.73m^2^. Proteinuria and hematuria were shown in various degrees because patients with various background kidney diseases were included in the study as described in Table 2.

**Table 1:** Clinical characteristics of the patients with or without central fibrous area in the glomerular vascular pole at the time of kidney biopsy

	Total (*n* = 101)	CFA (+) (*n* = 56)	CFA (−) (*n* = 45)	*p* value
Age (year), median (IQR)	62.5 (55.3–71.0)	67.0 (53.0–72.0)	51.5 (35.3–67.3)	0.0016
Gender (male/female, %)	51/50 (50.5)	29/27 (51.8)	22/23 (48.9)	0.77
Body mass index (kg/m^2^), median (IQR)	22.9 (20.6–26.0)	23.0 (20.5–26.1)	22.8 (21.1–24.8)	0.92
Hypertension, *n* (%)	56 (56.0)	38 (69.1)	18 (40.0)	0.0036
Diabetes mellitus, *n* (%)	23 (22.3)	15 (26.8)	8 (17.8)	0.28
Hyperlipidemia, *n* (%)	46 (47.4)	32 (58.2)	14 (33.3)	0.015
Hyperuricemia, *n* (%)	21 (22.3)	11 (20.8)	10 (24.4)	0.67
Proteinuria (g/g creatinine)				0.90
< 0.3, *n* (%)	13 (12.9)	7 (12.5)	6 (13.3)	
0.3–1.0, *n* (%)	29 (28.7)	17 (30.4)	12 (26.7)	
1.0–3.0, *n* (%)	19 (18.8)	10 (17.9)	9 (20.0)	
> 3.0, *n* (%)	40 (39.6)	22 (39.3)	18 (40.0)	
Hematuria (RBC /hpf)				0.34
< 5, *n* (%)	34 (33.7)	19 (33.9)	15 (33.3)	
5–20, *n* (%)	23 (22.8)	16 (28.6)	7 (15.6)	
20–100, *n* (%)	9 (8.9)	5 (8.9)	4 (8.9)	
> 100, *n* (%)	35 (34.7)	16 (28.6)	19 (42.2)	
Serum creatinine (mg/dL), median (IQR)	0.86 (0.66–1.17)	0.91 (0.67–1.19)	0.84 (0.62–1.15)	0.59
eGFR (ml/min/1.73m^2^), median (IQR)	63.5 (39.0–83.7)	58.2 (37.9–77.0)	68.8 (43.9–92.0)	0.15

*Abbreviations*: *CFA* Central fibrous area, *RBC* Red blood cell, *eGFR* Estimated glomerular filtration rate, *IQR* Interquartile range

**Table 2:** Histological characteristics of patients with or without central fibrous area in the glomerular vascular pole

	Total (*n* = 101)	CFA (+) (*n* = 56)	CFA (−) (*n* = 45)	*p* value
Number of CFA, *n*, median (IQR)	1 (0–3)	2 (2–3.25)	0 (0–0)	―
Number of glomeruli, *n*, median (IQR)	22 (16–31)	21 (15–31.75)	22 (18–30)	0.49
Global glomerulosclerosis, *n*, median (IQR)	1 (0–3)	1 (0–3.25)	1 (0–2)	0.58
CFA-positive ratio, %, median (IQR)	5.3 (0–12.5)	11.1 (8.3–17.9)	0 (0–0)	―
Segmental glomerulosclerosis, *n*, median (IQR)	0 (0–0)	0 (0–0)	0 (0–0)	0.97
Glomerular tuft size (μm^2^), median (IQR)	13,282 (10,808–16,797)	12,957 (10,893–16,834)	13,775 (10,786–16,632)	0.67
Elastofibrosis in the interlobular artery, *n* (%)	61 (63.5)	40 (75.5)	21 (48.8)	0.0022
Mild, *n* (%)	19 (19.8)	10 (18.9)	9 (20.9)	
Moderate, *n* (%)	12 (12.5)	9 (17.0)	3 (7.0)	
Severe, *n* (%)	30 (31.3)	21 (40.0)	9 (20.9)	
Arteriolar hyalinosis, *n* (%)	57 (56.4)	38 (71.7)	19 (42.2)	0.016
Mild, *n* (%)	42 (41.6)	28 (52.8)	14 (31.1)	
Moderate, *n* (%)	8 (7.9)	5 (9.4)	3 (6.7)	
Severe, *n* (%)	7 (6.9)	5 (9.4)	2 (4.7)	
Tubular atrophy, *n* (%)	80 (79.2)	52 (92.9)	38 (84.4)	0.30
Mild, *n* (%)	73 (72.3)	42 (75.0)	31 (68.9)	
Moderate, *n* (%)	13 (12.9)	7 (12.5)	6 (13.3)	
Severe, *n* (%)	4 (4.0)	3 (5.4)	1 (2.2)	
Interstitial fibrosis, *n* (%)	47 (46.5)	28 (50.0)	19 (42.2)	0.30
Mild, *n* (%)	31 (30.7)	17 (30.4)	14 (31.1)	
Moderate, *n* (%)	14 (13.9)	9 (16.1)	5 (11.1)	
Severe, *n* (%)	2 (2.0)	2 (3.6)	0 (0.0)	
Pathological Diagnosis				
Benign nephrosclerosis, *n* (%)	8 (7.9)	5 (8.9)	3 (6.7)	0.68
Mesangial proliferative GN, *n* (%)	26 (25.7)	12 (21.4)	14 (31.1)	0.27
IgA nephropathy, *n* (%)	20 (19.8)	8 (14.3)	12 (26.7)	0.12
IgA vasculitis, *n* (%)	2 (2.0)	1 (1.8)	1 (2.2)	0.88
Non-IgA mesangial proliferative GN, *n* (%)	4 (4.0)	3 (5.4)	1 (2.2)	0.42
Endocapillary proliferative GN, *n* (%)	2 (2.0)	1 (1.8)	1 (2.2)	0.88
Lupus nephritis, *n* (%)	7 (6.9)	3 (5.4)	4 (8.7)	0.40
ANCA-associated vasculitis, *n* (%)	9 (8.9)	6 (10.7)	3 (6.7)	0.48
Proteinuric glomerular diseases, *n* (%)	37 (36.6)	24 (42.9)	13 (28.9)	0.59
Minimal change disease, *n* (%)	7 (6.9)	2 (3.6)	5 (11.1)	0.14
Focal segmental glomerulosclerosis, *n* (%)	2 (2.0)	1 (1.8)	1 (2.2)	0.88
Membranous nephropathy, *n* (%)	21 (20.8)	16 (28.6)	5 (11.1)	0.03
Diabetic glomerulosclerosis, *n* (%)	7 (6.9)	5 (9.0)	2 (4.4)	0.38
Acute TIN/Acute tubular injury, *n* (%)	8 (7.9)	6 (10.8)	2 (4.4)	0.16
Minor glomerular abnormalities	4 (4.0)	3 (5.4)	1 (2.2)	0.42
Thrombotic microangiopathy	2 (2.0)	1 (1.8)	1 (2.2)	0.88
Thin basement membrane disease	2 (2.0)	1 (1.8)	1 (2.2)	0.88
Others, *n* (%)	5 (5.0)	0 (0.0)	5 (11.1)	0.01

*Abbreviations*: *CFA* Central fibrous area, *GN* Glomerulonephritis, *ANCA* Anti-neutrophil cytoplasmic antibody, *MCD* Minimal change disease, *FSGS* Focal segmental glomerulosclerosis, *MN* Membranous nephropathy, *DN* Diabetic nephropathy, *TIN* tubulointerstitial nephritis, *MGA* Minor glomerular abnormalities, *TMA* Thrombotic microangiopathy, *TBMD* Thin basement membrane disease, *IQR* Interquartile range. The group ‘Others’ contained focal glomerular obsolescence (*n* = 2), smoking-related glomerulopathy (*n* = 1), acute pyelonephritis (*n* = 1), and glomerular hypertrophy (*n* = 1)

**Fig. 1:**
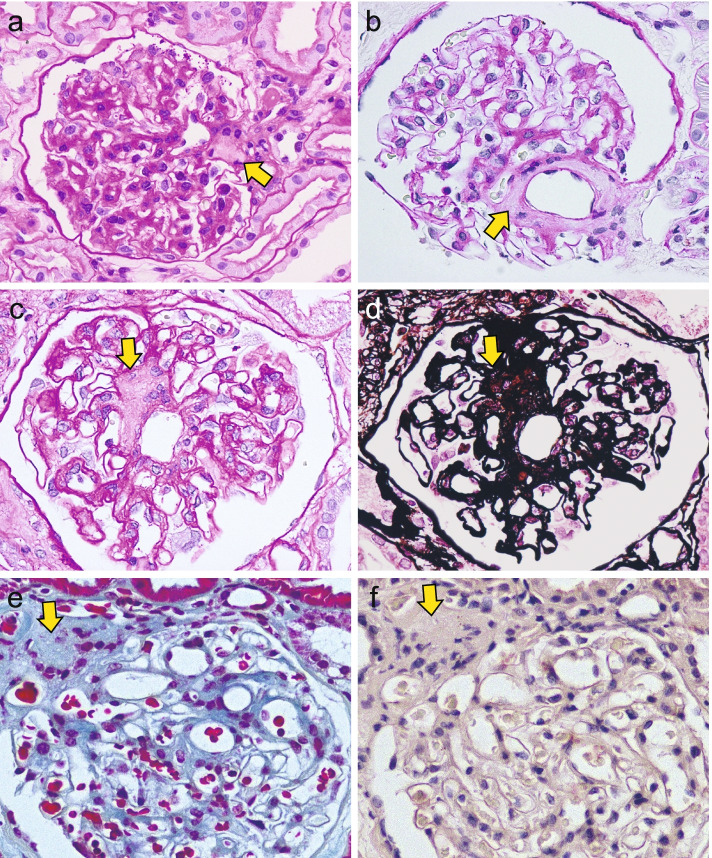
Histological findings of central fibrous area (CFA) in the glomerular vascular pole. **a** CFA was observed as a monotonous fibrous lesion in the glomerular vascular pole (arrow). **b** CFA was also found as a fibrous lesion encircling afferent arteriole (arrow). **c–e** The area was stained with periodic-acid Schiff weakly positive (**c**; arrow), periodic-acid silver methenamine positive (**d**; arrow), and blue in Masson’s-trichrome (**e**; arrow). **f** Congo red staining was negative (arrow), differing from renal amyloidosis. Magnification × 400

CFAs were observed in 56 of the 101 (55.4%) specimens. The histological characteristics of the CFAs were examined by histostaining, immunostaining, and electron microscopy. A CFA is a small monotonous accumulation located in the glomerular vascular pole that often encircles an afferent arteriole without compression (Fig. 1a and b). The staining pattern of CFA was PAS-weak positive, argyrophilic, and blue in trichrome (Fig. 1c-e). This staining pattern is distinct from that of the mesangial matrix. Congo red staining was negative for all samples (Fig. 1f).

**Fig. 2:**
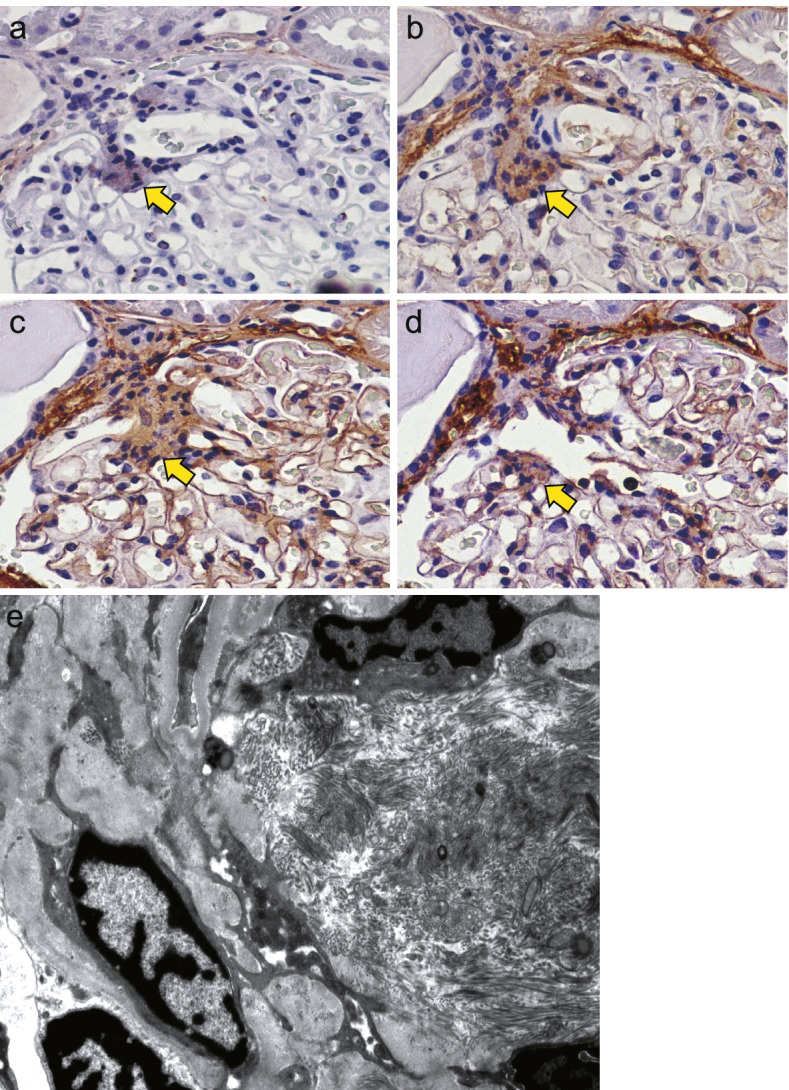
Central fibrosis area (CFA) consisted of fibrillar collagens in addition to matrix collagens. **a–d** Immunostaining for collagen I (**a**), collagen III (**b**), collagen IV (**c**), and collagen VI (**d**) (magnification × 400). Collagen III, one of the major fibrillar collagens, was present in CFA (arrows) in addition to non-fibrillar collagens, collagens IV and VI. Collagen I was weak-positive. **e** Electron microscopy showed enrichment of fibrous collagens (right side) in CFA, distinguishing them from mesangial matrix components in the mesangium (left side) (magnification × 6000)

Immunostaining revealed that CFAs consisted of fibrillar collagen (collagen III) in addition to non-fibrillar collagens (collagen IV and VI) (Fig. 2a-d). Collagen I was weakly positive in CFAs. Electron microscopy confirmed that CFAs consisted of fibril-rich fibers with bundles, indicating interstitial fibrillar collagens (Fig. 2e), differing from the non-fibrillar structure of the mesangial matrix.

**Fig. 3:**
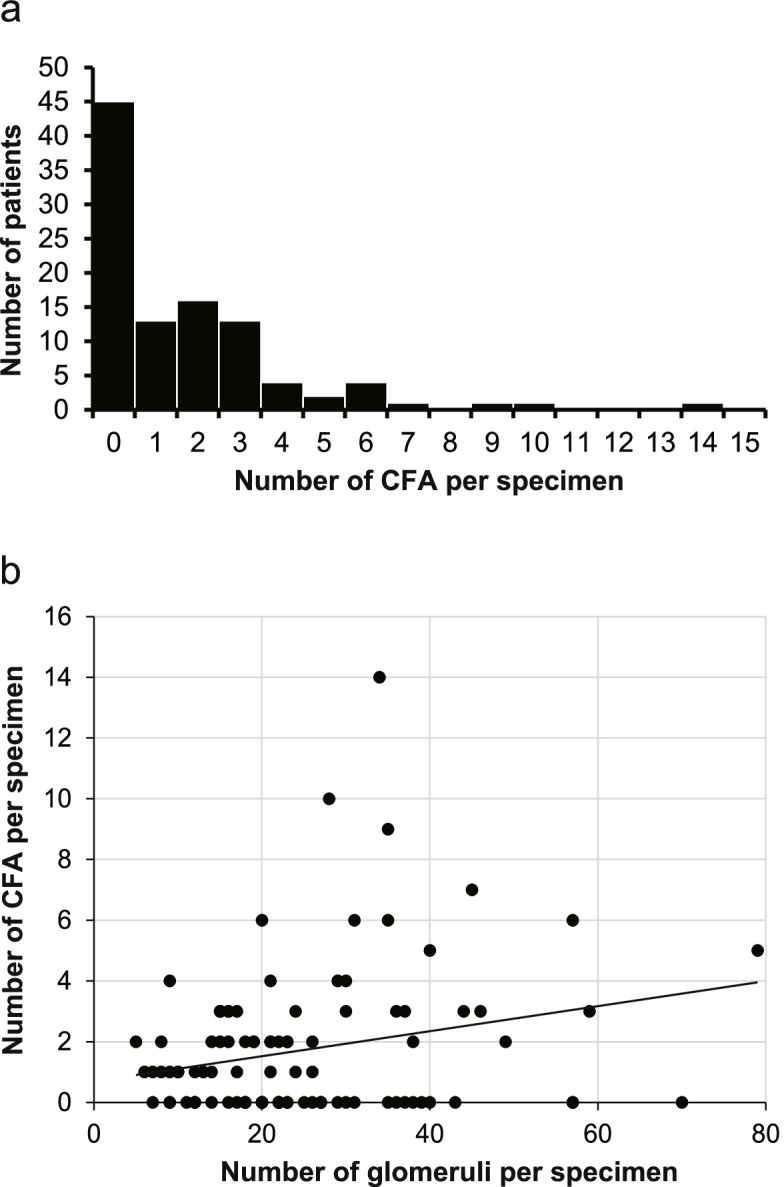
Distribution of the number of central fibrous areas (CFA) per specimen. **a** Histogram of the number of CFA per specimen. Most patients had from one to three CFAs, and some four or more. **b** The number of CFAs per specimen was significantly correlated with the number of glomeruli per specimen, although its correlation was quite low (R^2^ = 0.058, *p* = 0.015)

CFAs were detected in a median of two of 21 glomeruli in specimens containing CFAs (Table 2). The majority (42 cases; 75%) included three or fewer specimens, and the remaining 14 cases (25%) included four or more specimens (Fig. 3a). The median CFA-positive ratios were 5.3% [IQR 0–12.5] in all patients and 11.1% [IQR 8.3–17.9] in specimens containing CFAs (Table 2). CFA numbers were significantly correlated with glomerular numbers obtained; however, the correlation was quite low (R^2^ = 0.058, *p* = 0.015; Fig. 3b).

### Clinical and histological findings in relation to CFA formation

The patients were divided into two groups [CFA (+) or CFA (−)] according to the presence or absence of CFAs in the obtained samples. Subsequently, clinicopathological correlations with CFA formation were analyzed.

Clinically, the CFA (+) group was older [median (IQR) 67.0 (53.0–72.0) vs. 51.5 (35.3–67.3) years; *p* < 0.01] and had a significantly higher prevalence of hypertension (69.1 vs. 40.0%; *p* < 0.01) and hyperlipidemia (58.2% vs. 33.3%; *p* < 0.05) than the CFA (−) group (Table 1). No significant differences were noted in sex, body mass index, diabetes mellitus, hyperuricemia, proteinuria, microscopic hematuria, serum creatinine level, or estimated GFR. Histologically, elastofibrosis of the interlobular artery (75.5 vs. 48.8%; *p* < 0.01) and arteriolar hyalinosis (71.7% vs. 42.2%; *p* < 0.05) were significantly more frequent in the CFA (+) group than in the CFA (−) group (Table 2). There were no significant differences in the number of glomeruli showing global glomerulosclerosis and segmental glomerulosclerosis, glomerular tuft size, tubular atrophy, and interstitial fibrosis. With regard to background kidney diseases, membranous nephropathy was more frequent in the CFA (+) group than in the CFA (−) group (28.6% vs. 11.1%; *p* < 0.05), although a variety of kidney diseases included CFAs. For multivariate analysis, age, glomerular tuft size, serum creatinine level, diabetes mellitus, and hyperlipidemia were selected as the most significant factors associated with CFA formation among the clinical and histological findings by stepwise regression analysis (Table 3). Among them, higher age was the only independent factor associated with CFA formation (odds ratio 1.43 [95% CI 1.09–1.90), per 10 years; *p* = 0.011).

**Table 3:** Multivariate analysis to detect associated factors for central fibrous area formation in the glomerular vascular pole

	Odds ratio (95% CI)	*p* value
Age (per 10 years)	1.43 (1.09–1.90)	0.011
Glomerular tuft size	1.00 (1.00–1.00)	0.13
Serum creatinine	0.74 (0.45–1.22)	0.24
Hyperlipidemia	0.37 (0.13–1.01)	0.053
Diabetes mellitus	0.99 (0.29-–3.34)	0.99

*Abbreviations*: *CI* Confidence interval

Because membranous nephropathy was univariately more frequent in the CFA (+) group than the CFA (−) group, we compared clinicopathological characteristics between membranous nephropathy patients with and without CFA formation (Supplementary Tables 1 and 2). No significance between the two groups was observed, including for age.

### A few alpha-smooth muscle actin-positive cells exist in or adjacent to CFA

**Fig. 4:**
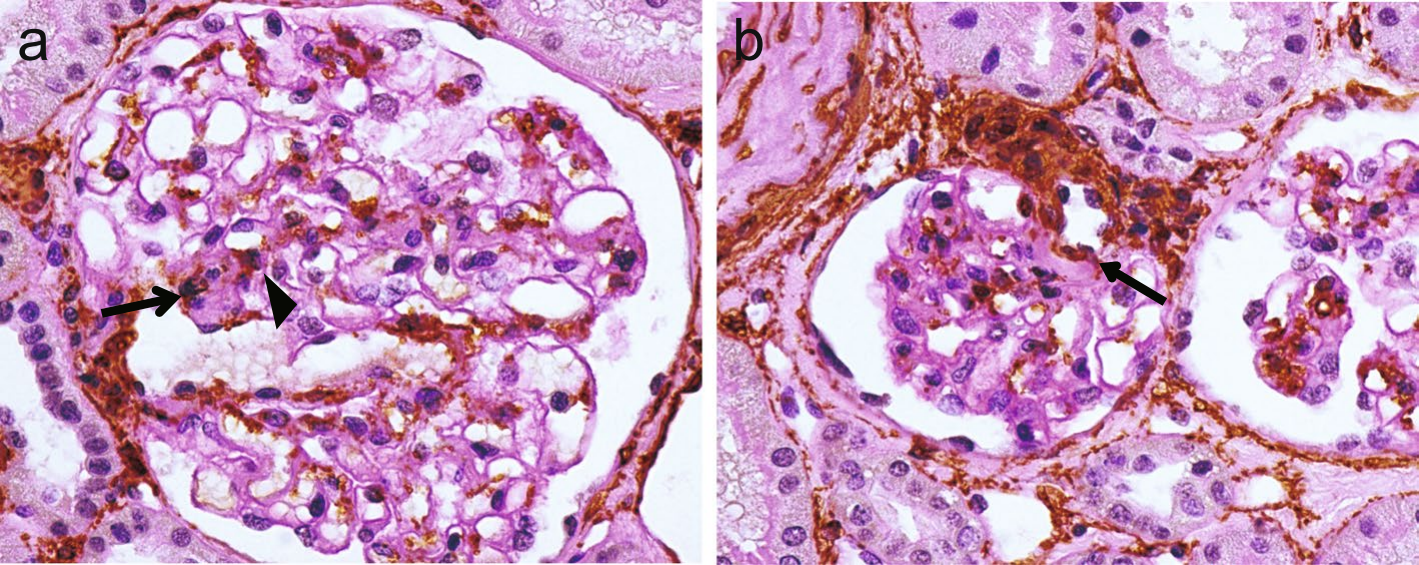
Immunostaining for alpha smooth-muscle actin (α-SMA). **a** α-SMA-positive cells were observed in the central fibrous area (CFA; arrow). The cells were also detected in the mesangial area adjacent to CFA (arrowhead). **b** α-SMA-positive cells were also observed in the capillary wall of afferent arteriole adjacent to CFA (arrow), while there were no such cells in the CFA. Magnification × 400. Periodic-acid Schiff staining was performed as a counterstaining

To determine the presence of myofibroblasts in CFAs, immunostaining for α-SMA was performed in 58 CFA-containing glomeruli from 26 patients. Twenty-seven of the 58 (46.6%) glomeruli contained α-SMA-positive cells in or adjacent to CFA (Fig. 4). Only five of these glomeruli had a few α-SMA-positive cells in CFAs, with the remaining glomeruli showing α-SMA-positive cells adjacent to CFAs. In contrast, glomeruli of cases without CFAs did not contain α-SMA-positive cells, except for mesangial and vascular smooth muscle cells (Supplementary Fig. 1).

## Discussion

A CFA is a monotonous, fibril-rich lesion located in the glomerular vascular pole. Its formation is associated with older age. Aging kidneys show various histological findings, including glomerulosclerosis, tubular atrophy, interstitial fibrosis, arteriosclerosis, and nephron loss [1–5, 17]. Although these structural changes cause GFR decline, they are non-specific and thus difficult to distinguish from secondary scar lesions induced by hypertension, diabetes mellitus, and primary kidney diseases. In the present study, although CFAs were observed in various kidney diseases and their formation was univariately associated with hypertension, hyperlipidemia, sclerotic vascular changes of the interlobular artery and arteriole, and membranous nephropathy, multivariate analysis revealed that higher age was the only independent factor associated with CFA formation. Our results confirmed that CFA could serve as a histological marker of older age.

CFA is observed in various kidney diseases and its frequency increases with age. Inomata first described this pathological finding as vascular pole deposits (VPD) [15]. VPD was observed in 8.9% of 2963 cases of various kidney diseases, and its number per specimen was 1 in most cases [15]. Inomata concluded that VPD was not disease-specific and had no bearing on age, sex, or any clinical signs and symptoms, including nephrotic syndrome and hypertension [15]; however, statistical analysis was not performed to support this conclusion. Recently, Kanetsuna et al. Showed that CFA significantly increased in older kidneys compared to younger kidneys, using zero-hour biopsy specimens from living renal donors, and IgA nephropathy biopsy specimens [14]. Although the frequency of CFA was not shown, the average number of CFAs per specimen was 3.0 of 49.7 glomeruli (6.0%) in the zero-hour biopsy group from living kidney donors and 1.2 of 19.9 glomeruli (6.0%) in the IgA nephropathy group [14]. In the present study, we detected CFA in 55.4% of cases, greater than the frequency in Inomata’s research, whereas the number of CFA per specimen was 2 and the CFA-positive rate was 5.3%, similar to the 2 previous studies [14, 15]. Using various kidney diseases, including IgA nephropathy, we also confirmed that older age was the only predictor associated with CFA formation, supporting the results of Kanetsuna et al. [14]. The difference in CFA frequency could be due to two reasons. First, the age distribution of the enrolled subjects differed among studies. Although Inomata’s study did not provide the average age, most participants were under 40 [15]. In Kanetsuna’s analysis, the mean age was 58.3 ± 11.2 years old in the zero-hour group and 33.1 ± 13.9 years old in the IgA nephropathy group [14]. Considering that the median age in the CFA (+) group in our study was 62.5 years old, the fact that the frequency in our study was more significant than those of Inomata’s research and the IgA nephropathy group in Kanetsuna’s study, and was similar to that of the zero-hour biopsy group in Kanetsuna’s study, was not unusual. Second, the definition of CFA differed slightly among studies. Inomata only included hyaline deposits as VPDs [15], whereas Kanetsuna et al. [14] included both hyaline and fibrous lesions. This study and Inomata’s included cylindrical shapes in addition to nodular forms [15], whereas Kanetsuna et al. included only nodular lesions [14]. Given the different age populations among these studies, these differences may be due to the stage of CFA formation. VPD may be a precursor of CFA and may be present in younger populations, and then CFA may form in advanced age. In summary, CFA is observed in various kidney diseases and is a helpful pathological finding for detecting kidney aging.

The morphogenesis of CFAs remains unresolved in the context of advanced age. Neal et al. Termed the glomerular vessels embedded in the mesangium of the vascular pole as the vascular chamber and showed that the vascular chamber widened in correlation with glomerular tuft size in the adult kidney [18]. Using multiphoton imaging and electron microscopy, they elegantly revealed that collagen bundles containing fibrous collagens appeared at the wall of the afferent vascular chamber [18], suggesting that the collagen bundles are formed by the hemodynamic effect that regulates glomerular pressure. In our study, immunohistochemistry revealed that CFA consisted of fibrillar collagens (collagen I and III), in addition to nonfibrillar collagens (collagen IV and VI). Electron microscopy also confirmed that the presence of fibrillar collagens in CFA is distinct from the extracellular matrix components of the mesangial matrix, which consist mainly of non-fibrillar collagens [19], supporting the findings of Neal’s study. Because vascular chambers are associated with large adult glomeruli [18], we speculate that longstanding high hemodynamic pressure on the walls of vascular chambers accelerates CFA formation in aged kidneys, as discussed by Kanetsuna et al. [14]. In addition, intrinsic glomerular cells, such as mesangial cells, vascular smooth muscle cells, pericytes, and endothelial cells of the afferent vascular chamber, might produce fibrillar collagens in the context of advanced age and kidney diseases. Under disease conditions, mesangial cells acquire a myofibroblast-like phenotype that produces interstitial collagens in addition to normal matrix constituents [20]. Potassium ion channels K (Ca)3.1 on mesangial cells, promote transforming growth factor β1-induced premature senescence and myofibroblast phenotype transition of mesangial cells [21]. These findings suggest a connection between cellular senescence and myofibroblast-like phenotypic changes in mesangial cells. Likewise, vascular smooth muscle cells, pericytes, and endothelial cells of the afferent vascular chamber may be associated with interstitial collagen production due to cellular senescence induced by aging, hemodynamic pressure, and kidney diseases. In our study, α-SMA-positive cells, a major marker of myofibroblasts [22], were observed in or adjacent to the CFA, adding weight to our speculation. Although the frequency of α-SMA-positive cell inclusion was relatively low, and other underlying mechanisms may also be implicated in the morphogenesis of CFA, the accumulation of fibrillar collagens produced by myofibroblasts may be one mechanism underlying CFA formation at the glomerular vascular pole with increasing age, hemodynamic pressure, and diseases. Further studies are needed to elucidate the underlying mechanisms.

Another interesting question is whether CFA progresses to glomerulosclerosis and causes kidney dysfunction. Uesugi et al. Showed 3D imaging of the vascular tree of aging kidneys and determined that arteriosclerosis of the distal interlobular artery contributed to glomerulosclerosis [23]. In our study, CFA formation was univariately associated with vascular sclerosis of both the interlobular artery and arteriole, but not with glomerular tuft size, glomerulosclerosis, or kidney dysfunction. In addition, none of these histological findings were significantly associated with CFA formation on multivariate analysis. Although CFA often encircles afferent arterioles or vascular chambers, the vascular lumens do not become narrow, and the glomerular tuft does not shrink or enlarge. Furthermore, membranous nephropathy was more frequent in the CFA (+) group than in the CFA (−) group, but no clinicopathological difference was observed between the two groups of membranous nephropathy. Similarly, Kanetsuna et al. did not find any correlation between CFA formation and serum creatinine, estimated GFR, or glomerular sclerosis rate in either the IgA nephropathy group or zero-hour biopsy group [14]. Thus, CFA does not yet appear to directly cause glomerulosclerosis or kidney dysfunction and this point should be elucidated by a large cohort study in the future given the cross-sectional designs of both our study and the previous study [14].

CFA could be a useful histological marker for kidney aging. Detrimental effects of kidney aging have been reported increasingly. Kidneys from aged patients after acute kidney injury exhibit tertiary lymphoid tissue, which accelerates local inflammation and leads to kidney dysfunction [13]. In addition, kidney allografts from aged patients are associated with delayed graft function and risk of acute rejection, which could be linked to poor graft survival [5, 11–13]. Although, future studies are clearly needed to clarify the relationship between CFA formation and phenomena which are related to kidney aging, such as tertiary lymphoid tissue, chronic kidney disease progression, and allograft survival, recognizing CFA formation in the kidney might lead to appropriate clinical management of elderly patients with chronic kidney diseases.

Our study has some limitations. First, the patients had heterogeneous background kidney diseases, leading to small sample sizes given the variety of diseases. Advanced age and various kidney diseases that induce renal structural damage can promote the accumulation of senescent cells [5, 24]. In Kanetsuna’s study, the effect of higher age on CFA formation was more pronounced in IgA nephropathy specimens than in zero-hour biopsy specimens [14]. Thus, the heterogeneity of kidney specimens may have affected CFA formation. Second, CFA is formed only in the glomerular vascular pole; therefore, the prevalence of CFA may also depend on the sample size. Although the correlation between CFA numbers and glomerular numbers was low, the effect of kidney biopsy specimens could not be eliminated and large samples, such as wedge biopsy and nephrectomy specimens would be more helpful to assess the precise estimation of CFA frequency. Nonetheless, each specimen contained a sufficient number of glomeruli, and the number did not differ significantly between the CFA (+) and CFA (−) groups. Furthermore, the number of CFAs per specimen did not differ significantly from that of other studies [14, 15], thus minimizing the possibility of sample error in this study. Third, we could not exclude selection bias. Although we collected all consecutive patients who did not meet exclusion criteria from multiple facilities, kidney specimens had been collected from seven hospitals in the Hokuriku region of Japan for 1 year. In addition, all patients enrolled in the present study were Japanese adults. Aging-related kidney scarring is much less marked in Japanese patients than in Caucasian and African-American patients [25]. Although research related to CFA was conducted in the United Kingdom [18], it remains unknown whether CFA formation occurs in all races. Finally, the cross-sectional design did not permit us to assess the causal effect of CFA formation on such factors as chronic kidney disease progression. Similarly, although it was not observed whether CFA formation had any clinicopathological effect on membranous nephropathy, the result could be due to the small number sample of patients. Large cohort studies are needed to clarify whether CFA has a detrimental effect on various kidney diseases and works as a useful histological marker for aging kidneys.

## Conclusions

A CFA is a monotonous, fibrous, collagen-rich area in the glomerular vascular pole and is associated with older age. CFAs are observed in various kidney diseases and could serve as a histological finding in aging kidneys. A prospective large cohort study using homogenous kidney specimens is needed to clarify the clinical significance and morphogenesis of CFA formation.

## Supplementary Information

**Additional file 1.** Raw data for the analyses of the present study.

**Additional file 2: Supplementary Fig. 1.** Immunostaining for alpha smooth-muscle actin (α-SMA) on glomeruli of cases without central fibrous area (CFA). α-SMA was positive in mesangial cells and vascular smooth muscle cells, which were also found in the CFA-containing glomeruli (magnification × 400). Periodic-acid Schiff staining was performed as a counterstaining.

**Additional file 3: Supplementary Table 1.** Clinicopathological characteristics of the membranous nephropathy patients with or without central fibrous area in the glomerular vascular pole at the time of kidney biopsy. **Supplementary Table 2.** Histological characteristics of the membranous nephropathy of patients with or without central fibrous area in the glomerular vascular pole at the time of kidney biopsy.

## Data Availability

All data generated or analyzed during this study are included in this published article and its supplementary information files.

## Abbreviations

CFA: Central fibrous area
GFR: Glomerular filtration rate
PAS: Periodic acid-Schiff
α-SMA: Alpha-smooth muscle actin
IQR: Interquartile range
