# Supplementary figures and images for: Central fibrous area in the glomerular vascular pole consists of fibrous collagens and is associated with advanced age: a cross-sectional study
Source: BMC Nephrol. 2022 Jun 11;23:204. doi: 10.1186/s12882-022-02835-2 (PMC9188109; doi:10.1186/s12882-022-02835-2)

S1 Figure

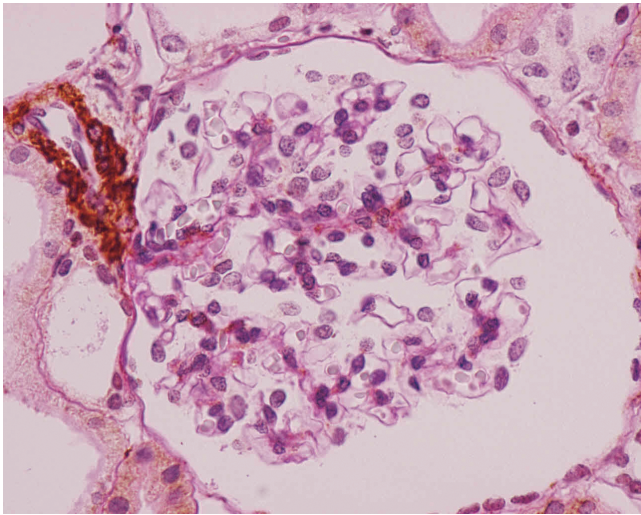

Supplement: Supplementary file 2 — Additional file 2: Supplementary Fig. 1. Immunostaining for alpha smooth-muscle actin (α-SMA) on glomeruli of cases without central fibrous area (CFA). α-SMA was positive in mesangial cells and vascular smooth muscle cells, which were also found in the CFA-containing glomeruli (magnification × 400). Periodic-acid Schiff staining was performed as a counterstaining. [file 12882_2022_2835_MOESM2_ESM.pdf]
