# Supplementary material for: Central fibrous area in the glomerular vascular pole consists of fibrous collagens and is associated with advanced age: a cross-sectional study
Source: BMC Nephrol. 2022 Jun 11;23:204. doi: 10.1186/s12882-022-02835-2 (PMC9188109; doi:10.1186/s12882-022-02835-2)
Supplement: Supplementary file 3 — Additional file 3: Supplementary Table 1. Clinicopathological characteristics of the membranous nephropathy patients with or without central fibrous area in the glomerular vascular pole at the time of kidney biopsy. Supplementary Table 2. Histological characteristics of the membranous nephropathy of patients with or without central fibrous area in the glomerular vascular pole at the time of kidney biopsy. [file 12882_2022_2835_MOESM3_ESM.docx]

Supporting Information

Supplementary Fig. 1 Immunostaining for alpha smooth-muscle actin (α-SMA) on glomeruli of cases without central fibrous area (CFA). α-SMA was positive in mesangial cells and vascular smooth muscle cells, which were also found in the CFA-containing glomeruli (magnification ×400). Periodic-acid Schiff staining was performed as a counterstaining.

Supplementary Table 1. Clinicopathological characteristics of the membranous nephropathy patients with or without central fibrous area in the glomerular vascular pole at the time of kidney biopsy

|  | Total (*n* = 21) | CFA (+) (*n* = 16) | CFA (-) (*n* = 5) | *p* value |
| --- | --- | --- | --- | --- |
| Age (year), median (IQR) | 71.0 (37.0–74.0) | 70.0 (64.8–75.0) | 71.0 (40.0–73.0) | 0.48 |
| Gender (male/female, %) | 12/9 (57.1) | 9/7 (56.3) | 3/2 (60.0) | 0.88 |
| Body mass index (kg/m^2^), median (IQR) | 23.5 (21.1–22.0) | 23.7 (22.0–25.6) | 21.6 (20.1–22.6) | 0.16 |
| Hypertension, *n* (%) | 15 (71.4) | 12 (75.0) | 3 (60.0) | 0.52 |
| Diabetes mellitus, *n* (%) | 6 (28.6) | 4 (25.0) | 2 (40.0) | 0.52 |
| Hyperlipidemia, *n* (%) | 16 (76.2) | 13 (81.3) | 3 (60.0) | 0.33 |
| Hyperuricemia, *n* (%) | 5 (23.8) | 5 (31.3) | 0 (0.0) | 0.15 |
| Proteinuria (g/g creatinine) |  |  |  | 0.58 |
| <0.3, *n* (%) | 0 (0.0) | 0 (0.0) | 0 (0.0) |  |
| 0.3-1.0, *n* (%) | 2 (9.5) | 2 (12.5) | 0 (0.0) |  |
| 1.0-3.0, *n* (%) | 1 (4.8) | 1 (6.3) | 0 (0.0) |  |
| >3.0, *n* (%) | 18 (85.7) | 13 (81.3) | 5 (100.0) |  |
| Hematuria (RBC /hpf) |  |  |  | 0.28 |
| <5, *n* (%) | 7 (33.3) | 6 (37.5) | 1 (20.0) |  |
| 5-20, *n* (%) | 12 (57.1) | 9 (56.3) | 3 (60.0) |  |
| 20-100, *n* (%) | 1 (4.8) | 1 (6.3) | 0 (0.0) |  |
| >100, *n* (%) | 1 (4.8) | 0 (0.0) | 1 (20.0) |  |
| Serum creatinine (mg/dL), median (IQR) | 0.77 (0.57–0.98) | 0.79 (0.65–1.03) | 0.71 (0.75–0.86) | 0.48 |
| eGFR (ml/min/1.73m^2^), median (IQR) | 74.0 (59.1–85.8) | 69.0 (56.6–81.2) | 83.0 (66.8–92.0) | 0.24 |

Abbreviations: CFA, central fibrous area; RBC, red blood cell; eGFR, estimated glomerular filtration rate; IQR, interquartile range

Supplementary Table 2. Histological characteristics of the membranous nephropathy of patients with or without central fibrous area in the glomerular vascular pole at the time of kidney biopsy

|  | Total  (*n* = 21) | CFA (+)  (*n* = 16) | CFA (-)  (*n* = 5) | *p* value |
| --- | --- | --- | --- | --- |
| Number of CFA, *n*, median (IQR) | 2 (1–3) | 2.5 (1–3) | 0 (0–0) | ― |
| Number of glomeruli, *n*, median (IQR) | 21 (16–30) | 22 (15.8–34.3) | 20 (18–22) | 0.54 |
| CFA-positive ratio, %, median (IQR) | 8.3 (4.1–13.3) | 9.3 (7.0–16.9) | 0 (0–0) | ― |
| Global glomerulosclerosis, *n*, median (IQR) | 2 (0–3) | 2 (1–3.25) | 0 (0–1) | 0.058 |
| Segmental glomerulosclerosis, *n*, median (IQR) | 0 (0–0) | 0 (0–0) | 0 (0–0) | >0.99 |
| Glomerular tuft size, µm^2^, median (IQR) | 15,045  (12,579–18,933) | 14,963  (11,399–17,443) | 16,285  (13,471–19,992) | 0.36 |
| Elastofibrosis in the interlobular artery, *n* (%) | 15 (71.4) | 11 (68.8) | 4 (80.0) | 0.59 |
| Mild, *n* (%) | 4 (19.0) | 3 (18.8) | 1 (20.0) |  |
| Moderate, *n* (%) | 3 (14.3) | 3 (18.8) | 0 (0.0) |  |
| Severe, *n* (%) | 8 (38.1) | 5 (31.3) | 3 (60.0) |  |
| Arteriolar hyalinosis, *n* (%) | 13 (61.9) | 11 (68.8) | 2 (40.0) | 0.65 |
| Mild, *n* (%) | 11 (52.4) | 9 (56.3) | 2 (40.0) |  |
| Moderate, *n* (%) | 1 (4.8) | 1 (6.3) | 0 (0.0) |  |
| Severe, *n* (%) | 1 (4.8) | 1 (6.3) | 0 (0.0) |  |
| Tubular atrophy, *n* (%) | 17 (81.0) | 14 (87.5) | 3 (60.0) | 0.36 |
| Mild, *n* (%) | 16 (76.2) | 13 (81.3) | 3 (60.0) |  |
| Moderate, *n* (%) | 0 (0.0) | 0 (0.0) | 0 (0.0) |  |
| Severe, *n* (%) | 1 (4.8) | 1 (6.3) | 0 (0.0) |  |
| Interstitial fibrosis, *n* (%) | 5 (23.8) | 5 (31.3) | 0 (0.0) | 0.36 |
| Mild, *n* (%) | 4 (19.0) | 4 (25.0) | 0 (0.0) |  |
| Moderate, *n* (%) | 0 (0.0) | 0 (0.0) | 0 (0.0) |  |
| Severe, *n* (%) | 1 (4.8) | 1 (6.3) | 0 (0.0) |  |

Abbreviations: CFA, central fibrous area; IQR, interquartile range.

Additional File. 1 Raw data for the analyses of the present study.
